# A Possible Role for WNT5A Hypermethylation in Pediatric Acute Lymphoblastic Leukemia

**DOI:** 10.4274/tjh.2013.0296

**Published:** 2015-05-08

**Authors:** Özden Hatırnaz Ng, Sinem Fırtına, İsmail Can, Zeynep Karakaş, Leyla Ağaoğlu, Ömer Doğru, Tiraje Celkan, Arzu Akçay, Yıldız Yıldırmak, Çetin Timur, Uğur Özbek, Müge Sayitoğlu

**Affiliations:** 1 İstanbul University Faculty of Medicine, Institute of Experimental Medicine Research (DETAE), Department of Genetics, İstanbul, Turkey; 2 İstanbul University İstanbul Faculty of Medicine, Department of Pediatric Hematology and Oncology, İstanbul, Turkey; 3 Akdeniz University Faculty of Medicine, Department of Pediatric Hematology and Oncology, Antalya, Turkey; 4 İstanbul University Cerrahpaşa Faculty of Medicine, Department of Pediatric Hematology and Oncology, İstanbul, Turkey; 5 Kanuni Sultan Süleyman Research and Training Hospital, Clinic of Pediatric Hematology and Oncology, İstanbul, Turkey; 6 Şişli Etfal Research and Training Hospital, Clinic of Pediatric Hematology and Oncology, İstanbul, Turkey; 7 Medeniyet University Faculty of Medicine, Göztepe Research and Training Hospital, Clinic of Pediatric Hematology and Oncology, İstanbul, Turkey

**Keywords:** WNT5A, Methylation, Downregulation, Gene expression, ALL

## Abstract

**Objective:**

WNT5A is one of the most studied noncanonical WNT ligands and is shown to be deregulated in different tumor types. Our aim was to clarify whether hypermethylation might be the cause of low WNT5A mRNA levels and whether we could restore this downregulation by reversing the event.

**Materials and Methods:**

The expression of WNT5A mRNA was studied in a large acute lymphoblastic leukemia (ALL) patient group (n=86) by quantitative real-time PCR. The methylation status was detected by methylation-specific PCR (MSPCR) and bisulphate sequencing. In order to determine whether methylation has a direct effect on WNT5A expression, disease-representative cell lines were treated by 5’-aza-20-deoxycytidine.

**Results:**

Here we designed a validation experiment of the WNT5A gene, which was previously examined and found to be differentially expressed by microarray study in 31 T-cell ALL patients. The expression levels were confirmed by quantitative real-time PCR and the expression levels were significantly lower in T-cell ALL patients than in control thymic subsets (p=0.007). MSPCR revealed that 86% of the patients were hypermethylated in the WNT5A promoter region. Jurkat and RPMI cell lines were treated with 5’-aza-20-deoxycytidine and WNT5A mRNA expression was restored after treatment.

**Conclusion:**

According to our results, WNT5A hypermethylation does occur in ALL patients and it has a direct effect on mRNA expression. Our findings show that epigenetic changes of WNT signaling can play a role in ALL pathogenesis and reversing methylation might be useful as a possible treatment of leukemia.

## Abstract (tr)

**Amaç:**

WNT5A, kanonik olmayan WNT ligandlarının en çok çalışılanıdır ve farklı tümör tiplerinde fonksiyon bozukluğu gösterdiği bilinmektedir. Amacımız tespit edilen düşük WNT5A mRNA miktarının altında yatan sebebin hiper metilasyon olup olmadığını açıklığa kavuşturmak ve bu düşüşü hiper metilasyonu tersine çevirerek düzeltip düzeltemeyeceğimizi belirlemekti.

**Gereç ve Yöntemler:**

WNT5A mRNA anlatımı, geniş bir ALL hasta gurubunda eş zamanlı kantitatif PZR ile çalışıldı (n=86). Metilasyon durumu metilasyona özgü PZR (MSPZR) ve bisülfit dizileme yöntemleri ile belirlendi. Metilasyonun WNT5A anlatımına doğrudan etkisi olup olmadığı ise hastalık özelliklerini gösteren hücre serilerine 5’-aza-20-deoxycytidine muamelesi ile gösterildi.

**Bulgular:**

Bu çalışmada daha önceki çalışmamızda mikro dizi analizi ile belirlenen düşük WNT5A anlatımının doğrulanması için mRNA anlatımı eş zamanlı kantitatif PZR yöntemi ile belirlendi ve T-ALL hastalarında kontrol timositlere göre istatistiki olarak anlamlı bir düşüş gözlendi (p=0,007). MSPZR ise hastaların %86’sında WNT5A geni promotör bölgesinin hiper metile olduğunu gösterdi. Jurkat ve RPMI hücre serileri 5’AZA ile muamele edildi ve WNT5A mRNA anlatımının yeniden arttığı belirlendi.

**Sonuç:**

Çalışmamızın sonuçlarına göre ALL hastalarında WNT5A hiper metilasyonu gözlenmektedir ve mRNA anlatımına doğrudan etkisi bulunmaktadır. Sonuçlarımız WNT sinyal ileti yolundaki epigenetik değişikliklerin ALL patogenezinde rol oynadığını göstermektedir ve metilasyonun tersine çevrilmesi lösemiler için olası bir tedavi yöntemi olarak kullanılabilir.

## INTRODUCTION

WNT signaling is a highly conserved pathway that has critical roles in differentiation, proliferation, migration, and hematopoiesis; it is widely studied in normal and malignant development [1]. The WNT pathway is divided into 2 main paths: the canonical, which is dependent on β-catenin, and the noncanonical, which is independent of β-catenin. The molecular mechanism of the canonical WNT pathway has been studied comprehensively, whereas the exact mechanism of the noncanonical WNT pathway remains unclear. Noncanonical pathways are activated by the binding of specific Wnt ligands like Wnt4, Wnt5a, and Wnt11 to Frizzled and Disheveled as in the canonical counterpart with different downstream members. Among all the noncanonical factors, WNT5A is most widely studied, since it was shown to have roles in both canonical and noncanonical WNT pathways. WNT5A was found to be overexpressed in many solid tumors, such as in breast, prostate, colon, and lung cancers [2,3,4,5]. On the contrary, it has been shown that WNT5A is essential for proper development but that hemizygous WNT5A mice could develop normally, except for the fact that some WNT5A hemizygous mice developed B-cell leukemia [6]. Liang et al. showed that WNT5A inhibits the proliferation of B-cells and plays a tumor-suppressing role in hematologic malignancies [7]. The role of canonical WNT signaling was also shown in normal T-cell development [8], but it is not well described in malignant development of T-cell leukemia. 

In our previous study, we showed that canonical WNT signaling is highly deregulated in T-acute lymphoblastic leukemia (ALL) patients. To evaluate the potential role of WNT signaling in T-cell leukemogenesis, we performed expression analysis of key components of the WNT pathway. More than 85% of the childhood T-ALL patients showed upregulated β-catenin expression at the protein level as compared to normal human thymocytes. The impact of this upregulation was reflected in high expression of known target genes (AXIN2, c-MYC, TCF1, and LEF). When the β-CATENIN gene was silenced by small interfering RNA, the cancer cells showed higher rates of apoptosis. These results demonstrate that abnormal WNT signaling activation occurs in a significant fraction of human T-ALL cases independently of known T-ALL risk factors. We concluded that deregulated WNT signaling is a novel oncogenic event in childhood T-ALL [9].

Here we show that WNT5A is downregulated in acute lymphoblastic leukemia patients, which is caused by epigenetic silencing. This finding confirms our previous results about abnormal canonical activation of WNT in T-ALL and the controversial actions of canonical and noncanonical WNT pathways.

## MATERIALS AND METHODS

### Patients and Controls

Eighty-six childhood ALL patients (52 males and 34 females) were included in this study. The mean age was 8.11 years (min: 16 days, max: 17 years). Bone marrow samples were obtained from patients at the time of diagnosis. All patients were treated according to the BFM protocol. The mean WBC count was 65x109/L (min: 1x109/L, max: 600x109/L) and cases were grouped as <50x109/L [n=45, mean: (129±110) x109/L] or >50x109/L [n=27, mean: (15±11)x109/L]. 

As controls, CD19-positive B-cells were obtained from the healthy bone marrow of transplantation donors (n=6) and normal peripheral blood (n=10), and healthy thymocytes were obtained from healthy thymus tissue of patients scheduled for cardiac surgery. The control samples were sorted with a FACS Aria II cell sorter (BD, USA). Mononuclear cells were isolated by density-gradient centrifugation over Ficoll-Hypaque and contained at least 90% B cells as determined by fluorescent CD19 staining for B-cell controls and CD3 for T-cell controls. This study was approved by the local ethics committee of the Medical Faculty of İstanbul University (reference number and date: 2008/305 and 20.02.2008) and informed consent was obtained from all patients and healthy controls or their families.

### RNA Isolation and cDNA Synthesis

Bone marrow samples were stored at -80 °C after homogenization in RTL buffer (QIAGEN, 79216). Total RNA was isolated with the QIAGEN RNeasy Protect Mini Kit (QIAGEN, 74104). RNA samples were treated with DNase (1 U/µg, Sigma, AMPD1-1KT) for possible DNA contaminations during isolation. One microgram of total RNA was used for cDNA synthesis by random hexamers and MMLV reverse transcriptase (MBI Fermentas, EPO351) according to the recommendations of the manufacturer. 

### Analysis of Gene Expression by Real-Time Quantitative RT-PCR

Quantitative PCR (QRT-PCR) was carried out on the Light Cycler Instrument 1.5 (Roche Diagnostics, Germany) with SYBR Premix Ex Taq (TAKARA, RR420A). The PCR conditions were prepared as per the instructions of the manufacturer and all samples were studied in duplicate. The specificity of amplification of the products was confirmed by melting curve analyses and agarose gel electrophoresis. The PCR program was as follows: initial denaturation at 95 °C for 7 min; amplification segment of 5 s at 95 °C, 10 s at 59 °C, and 10 s at 72 °C for 45 cycles; and melting curve segment of 15 s at 60 °C for 1 cycle. WNT5A relative expression levels were normalized to 3 reference genes (β-actin, CypA, and ABL).

### DNA Isolation, Bisulphate Treatment, Methylation-Specific PCR, and Bisulphate Sequencing

Following the DNA isolation, bisulphate treatment was performed as described previously [10]. Bisulphate-treated DNA was purified with the Gene Clean III Kit (Qbiogene, 74104) according to the manufacturer’s recommendations. After bisulphate treatment, the samples were amplified for the proximal region of the WNT5A promoter, which has been shown to regulate WNT5A transcription of the gene, by methylation-specific PCR (MSPCR). The MSPCR primers were designed by the MethPrimer database [11]. The PCR yields were analyzed on 4% agarose gel with ethidium bromide under UV light. As the MSPCR positive control, a healthy peripheral blood sample was methylated in vitro by the SssI methylase enzyme (New England Biolabs, M0226S). 

To confirm the MSPCR findings, samples that were treated with bisulphate were also sequenced directly by the Sanger sequencing method, with a primer set that was designed outside of the CpG island area. Sequence results were analyzed with the CLC Main Workbench 6.0 program as described by the manufacturer.

### WNT5A Gene Expression and 5-Azacytidine Treatments in Acute Leukemia Cell Lines

We analyzed WNT5A methylation and expression status among the acute leukemia cell lines (Fleb14-4, Molt4, Jurkat, T-ALL1, and RPMI 8402). Methylation was confirmed by bisulphate sequencing. The WNT5A methylated cell lines Jurkat and RPMI 8402 were cultured for 4 days with 5’-aza-20-deoxycytidine (Aza; Sigma-Aldrich, A3656) at 5 mM and 10 mM concentrations respectively in 24-well plates (5x105 cells/well) supplemented with 1 mL of RPMI 1640 medium with 10% fetal bovine serum (FBS) and 1% antibiotics (penicillin and streptomycin) at 37 °C in a humid atmosphere containing 5% CO2. At the 96th hour, the cells were collected for DNA and RNA isolations as previously described for MSPCR and QRT-PCR studies.

### Statistical Analysis

Relative expression levels were calculated by the delta Ct method. Differences between the relative expression levels of cases and controls were tested with the Mann-Whitney test. The clinical characteristics according to the methylation status were analyzed by chi-square test, Fisher’s exact test, or multivariate analysis where appropriate and p≤0.05 was considered statistically significant. The Kaplan-Meier method was used to estimate survival rates. The median follow-up was 66 months (min: 1 month, max: 158 months). Overall survival was defined by the interval from the date of diagnosis to the date of death or last follow-up. Event-free survival was defined as the time from diagnosis to treatment failure, relapse, death, or last follow-up. Differences were compared with the 2-sided log-rank test. Multivariate survival analysis was estimated according to the Cox regression model. All statistical analyses were done with SPSS 19.0 for Windows (IBM SPSS Data Editor Inc., USA) and GraphPad Prism V (GraphPad Software Inc., USA).

## RESULTS

### Downregulated Expression of WNT5A in Acute Lymphoblastic Leukemia Cases

**Figure 1:**
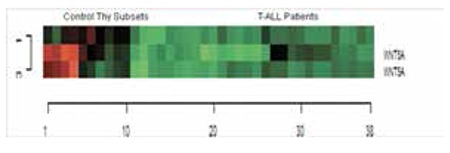
The heat map diagram of WNT5A probes in T-ALL patients and controls. Gene and samples were clustered using Euclidean distance and complete linkage method for the probe sets in 31 T-ALL patients and thymocyte subsets as controls [CD4 single positive, CD8 single positive, CD4+CD8+ double positive, Thymus (total thymus tissue), DP3- (CD4+, CD8+ double positive CD3 negative, immature single positive and CD3-, CD4-, CD8-)]. Three probe sets for WNT5A are illustrated in the heat map analysis. Green color shows downregulation and red color shows upregulation of the targeted gene.

The raw data of our previous expression array study [microarray data are available at http://www.ncbi.nlm.nih.gov/geo/ (accession no. GSE46170)] were reanalyzed and evaluated for the noncanonical WNT pathway members by defining a gene list obtained from public databases and web portals (NCBI Entrez Gene, WNT Homepage, http://www.stanford.edu/~rnusse/wntwindow.html). The data set was filtered to exclude genes showing minimal variation across the set of arrays from the analysis. Probe sets whose expression differed by at least 2-fold from the median in at least 20% of the arrays were retained. The heat map results showed that the WNT5A is downregulated in T-ALL patients when compared to control thymic subsets (Figure 1). 

**Figure 2:**
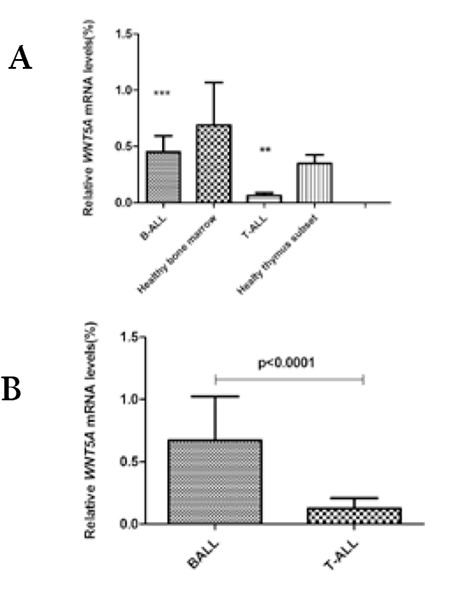
Relative WNT5A mRNA expression in B- and T-cell acute lymphoblastic leukemia patients. A) B-ALL patients’ samples were compared with healthy bone marrow samples and T-cell acute lymphoblastic leukemia patients’ were compared with healthy thymocytes (p=0.007). Each sample was studied in duplicate and threshold cycle numbers are relative to the geometric mean of 3 reference genes (ABL, β-actin, and CypA). B) Comparison of WNT5A mRNA levels between B-ALL and T-ALL samples (p<0.0001). Each sample was studied in duplicate and threshold cycle numbers are relative to the geometric mean of 3 reference genes (ABL, β-actin, and CypA).

To validate the downregulation of WNT5A detected in expression array analysis, quantitative mRNA expression of WNT5A was assessed by QRT-PCR for ALL patients’ samples. The expression of WNT5A was found to be downregulated both in B-ALL and T-ALL patients when compared to specific control cell populations (Figure 2A), but only in T-ALL was the difference statistically significant (p=0.007). Among the phenotypic groups, WNT5A mRNA levels were lower in T-ALL patients than B-ALL patients and the difference was statistically significant (Figure 2B, p<0.0001). 

### WNT5A Promoter is Heavily Methylated in Acute Lymphoblastic Leukemia Patients

**Figure 3:**
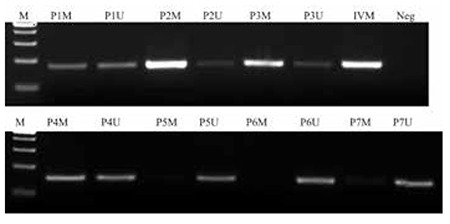
MSPCR results in acute lymphoblastic leukemia patients. The samples were run on 4% agarose gel with ethidium bromide under UV light. PCR products were 178 bp long. Initial M: pUC Mix Marker 8, P: patient, M: methylated PCR, U: unmethylated PCR (patients 1, 2, 3, and 4 are methylated; patient 7 is slightly methylated; patients 5 and 6 are unmethylated), IVM: in vitro methylated control sample, Neg: negative control.

**Figure 4:**
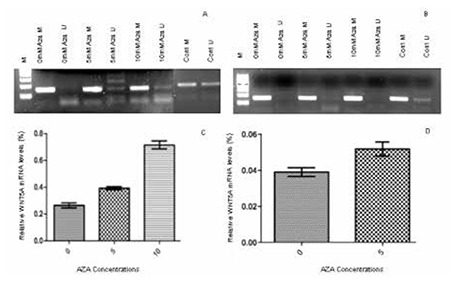
Aza-20-deoxycytidine (Aza) treatment experiments in cell lines. Cell lines Jurkat and RPMI 8402 were cultured for 4 days with Aza (Sigma-Aldrich, A3656) at 5 mM and 10 mM concentrations respectively in 24-well plates (5x105 cells/well) supplemented with 1 mL of RPMI 1640 medium with 10% FBS and 1% antibiotics (penicillin and streptomycin) at 37 °C in a humid atmosphere containing 5% CO2. A) MSPCR results after 0 mM, 5 mM, and 10 mM 5’-Aza treatment of RPMI 8402 cell line. B) MSPCR results after 0 mM, 5 mM, and 10 mM 5’-Aza treatment of Jurkat cell line. C) Relative WNT5A mRNA expression level after 5’-Aza treatment of RPMI 8402 cell line (5 mM, p=0.0043; 10 mM, p=0.0002). D) Relative WNT5A mRNA expression level after 5’-Aza treatment of Jurkat cell line (5 mM, p=0.004).

**Supplemental Figure 1:**
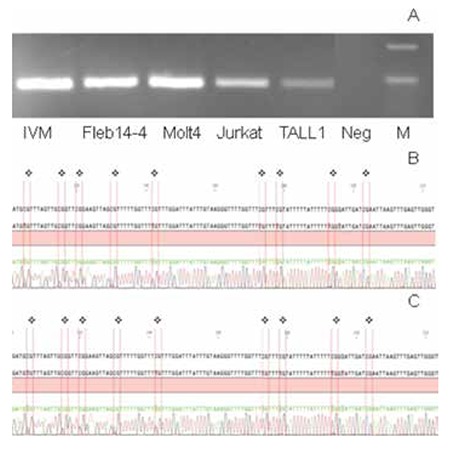
CpG island methylation status by bisulphate sequencing in ALL-derived cell lines. A) MSPCR results of cell lines, IVM: in vitro methylated control; B) bisulphate sequencing of Jurkat cell line, red bars show CpG islands; C) bisulphate sequencing of Fleb14-4 cell line, red bars show CpG islands.

According to MS-PCR results, in total 84% of the ALL patients were methylated for the WNT5A promoter region (Figure 3). Among the analyzed cell lines, Fleb14-4, Molt4, Jurkat, and T-ALL1 were determined as methylated for the WNT5A promoter region and the results were also confirmed by bisulphate sequencing, both in patients and cell lines (Supplemental Figure 1). To determine the effect of this methylated region on WNT5A transcription, we treated cell lines that were also methylated for the WNT5A promoter with Aza. In the RPMI 8402 cell line 10 mM (Figures 4A and 4C, p=0.0002) and in the Jurkat cell line 5 mM (Figures 4B and 4D, p=0.004) concentrations of Aza were able to demethylate the WNT5A promoter region and the WNT5A mRNA levels were increased after treatment, which shows that the studied region regulates WNT5A transcription.

### Clinical Implications of WNT5A Methylation

**Figure 5:**
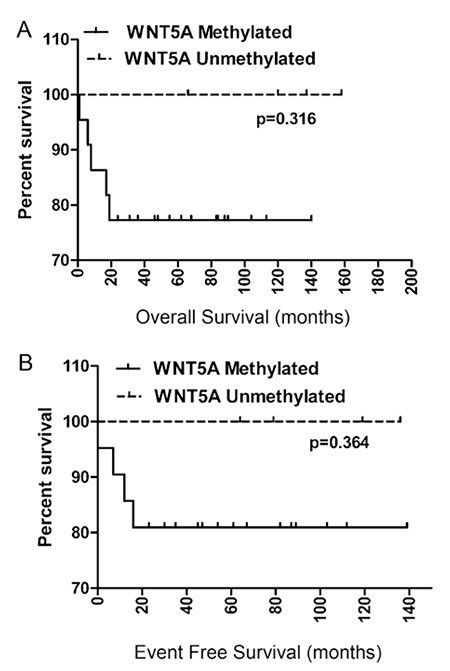
Kaplan-Meier estimate of probability of A) overall and B) event-free survival analysis.

The relations between WNT5A promoter methylation and clinical findings like age (p=0.50), sex (p=0.8), white blood cell count at the time of diagnosis (p=0.55), and immunophenotype (p=0.72) were analyzed and no statistical significance was detected; the results are summarized in Table 1. The Kaplan-Meier estimate of probability both for overall (p=0.31) and event-free (p=0.36) survival according to WNT5A methylation status showed no significant difference (Figures 5A and 5B, respectively). Multivariate regression analysis revealed no significant association between clinical features and WNT5A promoter methylation.

## DISCUSSION

WNT5A is one of the most studied noncanonical WNT ligands and it has shown to be expressed both in normal B and T cells [7,12]. WNT5A is found overexpressed in different solid tumors, such as in lung cancer, prostate cancer, metastatic carcinomas, and squamous head and neck carcinomas [13,14]. WNT5A was also described as a potential tumor suppressor gene, able to prevent and reverse tumor genesis [7,15]. However, like in endometrial carcinomas, WNT5A mRNA expression was downregulated in some malignant tumors [16,17] and types of leukemia [18,19,20]. One of the most well-known mechanisms for downregulation of a gene is epigenetic modification, such as promoter hypermethylation. WNT5A is silenced in most colorectal cancer cell lines and nasal NK/T-cell lymphoma due to promoter methylation [21].

Multistep deregulation of the WNT pathway was previously shown in childhood ALL by others and by our group [7,9,18]. In this study, we detected downregulation of WNT5A gene expression in T-ALL samples by microarray and confirmed decreased mRNA levels by real-time PCR both in B-ALL and T-ALL patients. The downregulation was more dramatic in T-ALL than B-ALL patients that had approximately 4-fold higher WNT5A mRNA levels. We then showed that downregulation of WNT5A expression is caused by promoter hypermethylation, which was previously described by Gomez et al., although their methylation rates were not as high as in our study [20]. This difference may be due to the primer regions that were selected. In this study, the primers were designed from the WNT5A proximal promoter, which resides within 631 bp upstream of the major transcription start site that was shown to have the strongest promoter activity [22]. The direct effect of the methylated region on transcription was confirmed by 5’-aza treatment, and when the methylation was undone, WNT5A expression was restored. Deng et al. also showed downregulation of WNT5A mRNA expression caused by promoter methylation, and it was restored in complete remission samples [23]. Some demethylating agents are also used in chemotherapy protocols and this may be the reason for loss of methylation in remission patients. The lack of remission samples limits our study as we could not detect the methylation status of remission samples, but we can speculate that the loss of WNT5A may cause leukemogenesis and methylation restoration might help complete remission in patients with leukemia. 

Hypermethylation of WNT5A has been reported previously both in T-ALL and B-ALL, but without any significant differences between the lineages. WNT5A was shown to be essential for normal T-cell development and the thymocytes of WNT5A knockout mice could rescue them from apoptosis [18], whereas the same effect could not be detected for B cells [7]. That result shows a role for noncanonical WNT signaling in cell death and T-cell homeostasis. Our results are in concordance with this finding, showing that the noncanonical pathway is downregulated specifically in T-ALL but not in B-ALL. This downregulation may cause the thymocytes to escape apoptosis and take part in leukemia development in our T-ALL cohort. Topol et al. showed that even low levels of WNT5A were able to reduce canonical WNT signaling via degradation of β-catenin, independent of GSK-3β activity [24].

In conclusion, we have demonstrated that WNT5A, an important member of the noncanonical WNT pathway, was silenced by hypermethylation in ALL. We have also shown that this methylation has a direct effect on gene expression, which may reveal that WNT5A can be used as a specific marker to reverse leukemic development.

## Figures and Tables

**Table 1:**
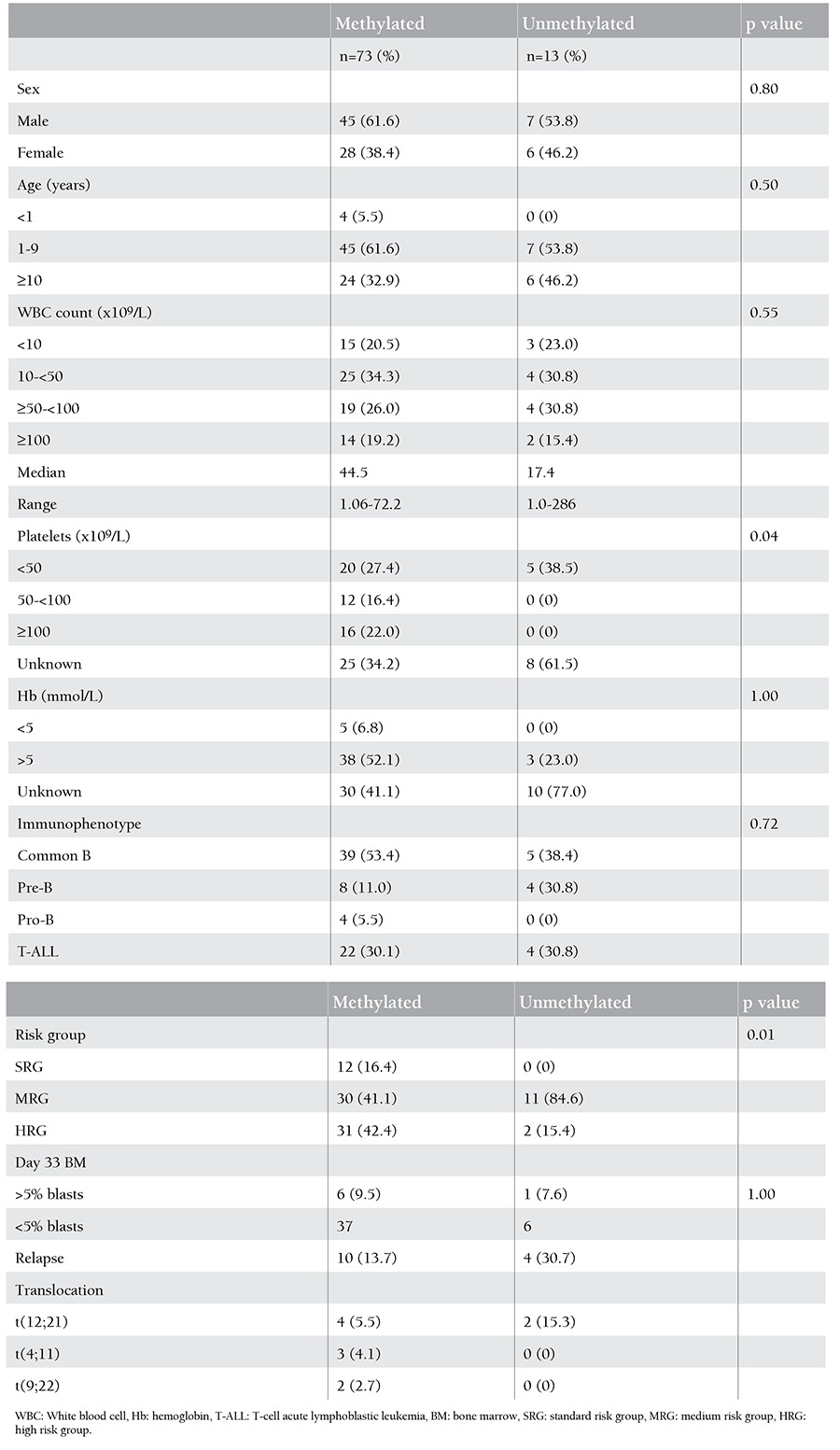
Clinical characteristics of pediatric acute lymphoblastic leukemia patients according to WNT5A methylation status.
